# Retrieving rice (*Oryza sativa* L.) net photosynthetic rate from UAV multispectral images based on machine learning methods

**DOI:** 10.3389/fpls.2022.1088499

**Published:** 2023-01-25

**Authors:** Tianao Wu, Wei Zhang, Shuyu Wu, Minghan Cheng, Lushang Qi, Guangcheng Shao, Xiyun Jiao

**Affiliations:** ^1^ College of Agricultural Science and Engineering, Hohai University, Nanjing, China; ^2^ State Key Laboratory of Hydrology-Water Resources and Hydraulic Engineering, Hohai University, Nanjing, China; ^3^ Cooperative Innovation Center for Water Safety and Hydro Science, Hohai University, Nanjing, China; ^4^ Jiangsu Key Laboratory of Crop Genetics and Physiology/Jiangsu Key Laboratory of Crop Cultivation and Physiology, Agricultural College, Yangzhou University, Yangzhou, China

**Keywords:** UAV multispectral remote sensing, rice canopy, net photosynthetic rate, vegetation index, textural index, machine learning

## Abstract

Photosynthesis is the key physiological activity in the process of crop growth and plays an irreplaceable role in carbon assimilation and yield formation. This study extracted rice (*Oryza sativa* L.) canopy reflectance based on the UAV multispectral images and analyzed the correlation between 25 vegetation indices (VIs), three textural indices (TIs), and net photosynthetic rate (Pn) at different growth stages. Linear regression (LR), support vector regression (SVR), gradient boosting decision tree (GBDT), random forest (RF), and multilayer perceptron neural network (MLP) models were employed for Pn estimation, and the modeling accuracy was compared under the input condition of VIs, VIs combined with TIs, and fusion of VIs and TIs with plant height (PH) and SPAD. The results showed that VIs and TIs generally had the relatively best correlation with Pn at the jointing–booting stage and the number of VIs with significant correlation (*p*< 0.05) was the largest. Therefore, the employed models could achieve the highest overall accuracy [coefficient of determination (*R* ^2^) of 0.383–0.938]. However, as the growth stage progressed, the correlation gradually weakened and resulted in accuracy decrease (*R* ^2^ of 0.258–0.928 and 0.125–0.863 at the heading–flowering and ripening stages, respectively). Among the tested models, GBDT and RF models could attain the best performance based on only VIs input (with *R* ^2^ ranging from 0.863 to 0.938 and from 0.815 to 0.872, respectively). Furthermore, the fusion input of VIs, TIs with PH, and SPAD could more effectively improve the model accuracy (*R* ^2^ increased by 0.049–0.249, 0.063–0.470, and 0.113–0.471, respectively, for three growth stages) compared with the input combination of VIs and TIs (*R* ^2^ increased by 0.015–0.090, 0.001–0.139, and 0.023–0.114). Therefore, the GBDT and RF model with fused input could be highly recommended for rice Pn estimation and the methods could also provide reference for Pn monitoring and further yield prediction at field scale.

## 1 Introduction

Photosynthesis is one of the most crucial parts of the global carbon and energy cycle (Reichstein et al., 2013; A Ivlev, 2017). The crop photosynthesis activities assimilate carbon dioxide (CO_2_) and water (H_2_O) by using light energy to form organic matter and, therefore, are a key determinant of food production and security (Reich and Amundson, 1985; Long et al., 2006). Net photosynthetic rate (Pn) is the value of the total photosynthetic rate minus the respiration rate, which directly refers to the organic matter accumulated. Although researchers have gradually deepened the understanding of photosynthesis based on cell-scale gas exchange, current methods and equipment developed based on these theories are still mainly focused on the leaf level, which is time-consuming and has a poor representation (Stinziano et al., 2019). It is scientific to use large canopy photosynthesis and transpiration measurement system (CAPTS) (Song et al., 2016) to observe photosynthesis at the canopy scale, but the investment is too expensive to be popularized in regional-scale monitoring.

The mobile high-throughput phenotyping platforms (HTPPs) (Deery et al., 2014; Li et al., 2014) with RGB, fluorescence, hyperspectral, thermal, 3D laser, and computed tomography (CT) imaging sensors provide a non-destructive method for rapid crop phenotypic acquisition. In particular, a high-spectral-resolution spectroradiometer (Aguirre-Gomez et al., 2001; Meroni and Colombo, 2006) (most Fieldspec 4 or 4pro, Analytical Spectral Devices, ASD, Boulder, CO, USA) is the most physical and effective equipment for photosynthesis monitoring on the ground. The sensitive band reflectance or vegetation indices (VIs), generally including 2 or more band reflectance, was commonly used to establish a linear or nonlinear relationship with crop physiological and biochemical parameters. Qiu et al. (2015) comprehensively analyzed the correlation between main photosynthetic, fluorescence parameters and hyperspectral data in ear position leaves of maize and found that Dλ699 had the best correlation with Pn. Sun et al. (2016) introduced wavelet analysis (WA) to select the sensitive bands of hyperspectral for estimating Pn of winter wheat on the leaf scale and found that the models based on WA were more accurate than the VIs method. Fu et al. (2019) constructed a stacking framework for retrieving the maximum carboxylation rate of Rubisco (V_c,max_) and the maximum electron transport rate supporting RuBP regeneration (J_max_) in the photosynthesis parameters of tobacco based on canopy hyperspectral reflectance, which further improve model accuracy compared with the basic models. Based on the advantages of ground platform on high-resolution continuous spectrum and texture features, the above research could provide practical and accurate estimation of photosynthetic parameters. However, the photosynthetic monitoring in the actual production field could hardly be represented due to environmental factors and the use of various equipment requires expertise.

As a new near-ground remote sensing approach, unmanned aerial vehicles (UAVs) (de Castro et al., 2021) can flexibly provide higher-resolution and bigger-scale images by carrying different sensors (e.g., multispectral, hyperspectral, and thermal infrared cameras). It has already been widely used in the inversion of physiological and biochemical parameters such as plant height (PH) (Che et al., 2020), leaf area index (LAI) (Chen et al., 2022b), nutrient states (Xu et al., 2021), and aboveground biomass (Wang et al., 2022). Equipped with hyperspectral imaging (HSI) sensors, Liu and Peng. (2020) employed eight chlorophyll-related VIs for estimating maximum Pn, and proposed a model based on chlorophyll index (CI) and photosynthetically active radiation (PAR) for different rice varieties. However, the water vapor in the field (especially the paddy field) might have a great influence on the hyperspectral data. Additionally, high price and tedious data processing process (e.g., noise processing, dimension reduction, and spectral unmixing) have prevented the commercial application of this method. Therefore, multispectral sensors that can characterize key points (usually including blue, green, red, red edge, and near-infrared bands) in crop canopy spectra features are more commonly used for practical application on UAV. Chen et al. (2018) established linear inversion models of photosynthetic parameters at different time points in the cotton bud stage based on UAV six-band multispectral images. However, cross-growth stage comparison and regression model selection could be done more comprehensively. Based on the relationship between VIs constructed from UAV multispectral image and photosynthetic parameters, Zhang et al. (2020a) explored the inversion method of diurnal variation of photosynthesis in rice canopy combined with the light response curve model and provided a method with physical basis for gross primary productivity (GPP) inversion, while the scale effect between 100-m UAV multispectral data and PAR monitoring data from a single point on the ground should be further discussed. On the other hand, the image obtained by UAV remote sensing has a higher resolution than satellite remote sensing; thus, it has more detailed texture features that can better reflect the difference in the set window size. Therefore, textural indices (TIs) are commonly introduced with VIs to improve the model accuracy. According to previous studies, TIs have a good correlation with aboveground biomass (Sarker and Nichol, 2011; Liu et al., 2019) and thus also have a good relationship with the accumulated amount of canopy elements (Pimstein et al., 2011; Lu et al., 2019; Zhang et al., 2021) (e.g., nitrogen, potassium, and chlorophyll). Zheng et al. (2019) have found that the normalized difference texture index (NDTI) is in good relationship with rice biomass and the fusion of NDTI with VIs improved the accuracy of biomass estimation. Similarly, Lu et al. (2021) and Zheng et al. (2020) demonstrated that the fusion of TIs and multispectral VIs could effectively improve the estimation of potassium accumulation and nitrogen accumulation in rice. Since the accumulated organic matter of photosynthesis can directly affect the basic growth indicators of rice such as plant height, tiller number, and leaf area index, TIs could also have untapped potential in Pn estimation.

For models employed in the inversion studies, linear regression or nonlinear regression were commonly used to construct inverse functions with definite expressions, but the accuracy is relatively low and poor in portability (Wan et al., 2021). Machine learning methods have been widely used in the regression and classification issues and have been proven to be fast, accurate, and good at generalization. WA (Bruce et al., 2002), partial least square regression (PLSR) (Fu et al., 2022), and least absolute shrinkage and selection operator (LASSO) (Yang and Bao, 2017) are usually used in HSI studies to reduce the high-dimension hyperspectral data to a few important components that are sensitive to the target parameters. Artificial neural network (ANN) (Liang et al., 2015), kernel-based support vector machine regression (SVR) (Liang et al., 2016), and random forest (RF) (Cheng et al., 2022) regression methods have been most wildly employed to explore and fit the nonlinear relationship between reflectance or VIs and inversion objects. Other machine learning methods based on ANN, kernel function, and tree also have great potential in this issue. Yang et al. (2022) built a Bayesian neural network (BNN) model to predict potential maximum quantum yield (Fv/Fm) and two other chlorophyll fluorometer parameters of grape by quantifying the HSI response indices of photosynthetic pigments and water status parameters. Yuan et al. (2022) simulated the maximum carboxylation rate at 25°C (V_m25_) of crops over time based on the convolutional neural network (CNN) model combining flux and satellite remote sensing data to further improve the estimation accuracy of GPP. Based on the leaf phenotype data, Zhang et al. (2020b) established poplar Pn estimating models using the extreme gradient boosting model (XGBoost). (Fu et al., 2020, 2022) have also proven the good performance of machine learning models based on the rich feature input of the HSI data for photosynthetic parameter estimation. However, there is a lack of understanding of machine learning methods for photosynthesis parameters estimating with less reflectance features based on the multispectral data and less research on rice.

In this study, multispectral images of rice canopy were acquired by UAV, and the responses of multispectral reflectance features together with Pn, PH, and SPAD to the different nitrogen or leakage treatments were analyzed at different growth stages. The correlation between Pn, VIs, and TIs extracted from the multispectral reflectance was compared, and the VIs with relatively significant correlations were employed as input of the five machine learning models. Model performance comparison under different input combinations was performed, and the improvement of fusing TIs and basal growth index PH and SPAD was further analyzed. The final purpose is to explore an economical and accurate method at field scale for the estimation of Pn and photosynthesis stress detection during the whole growth season of rice.

## 2 Materials and methods

### 2.1 Study area

**Figure 1:**
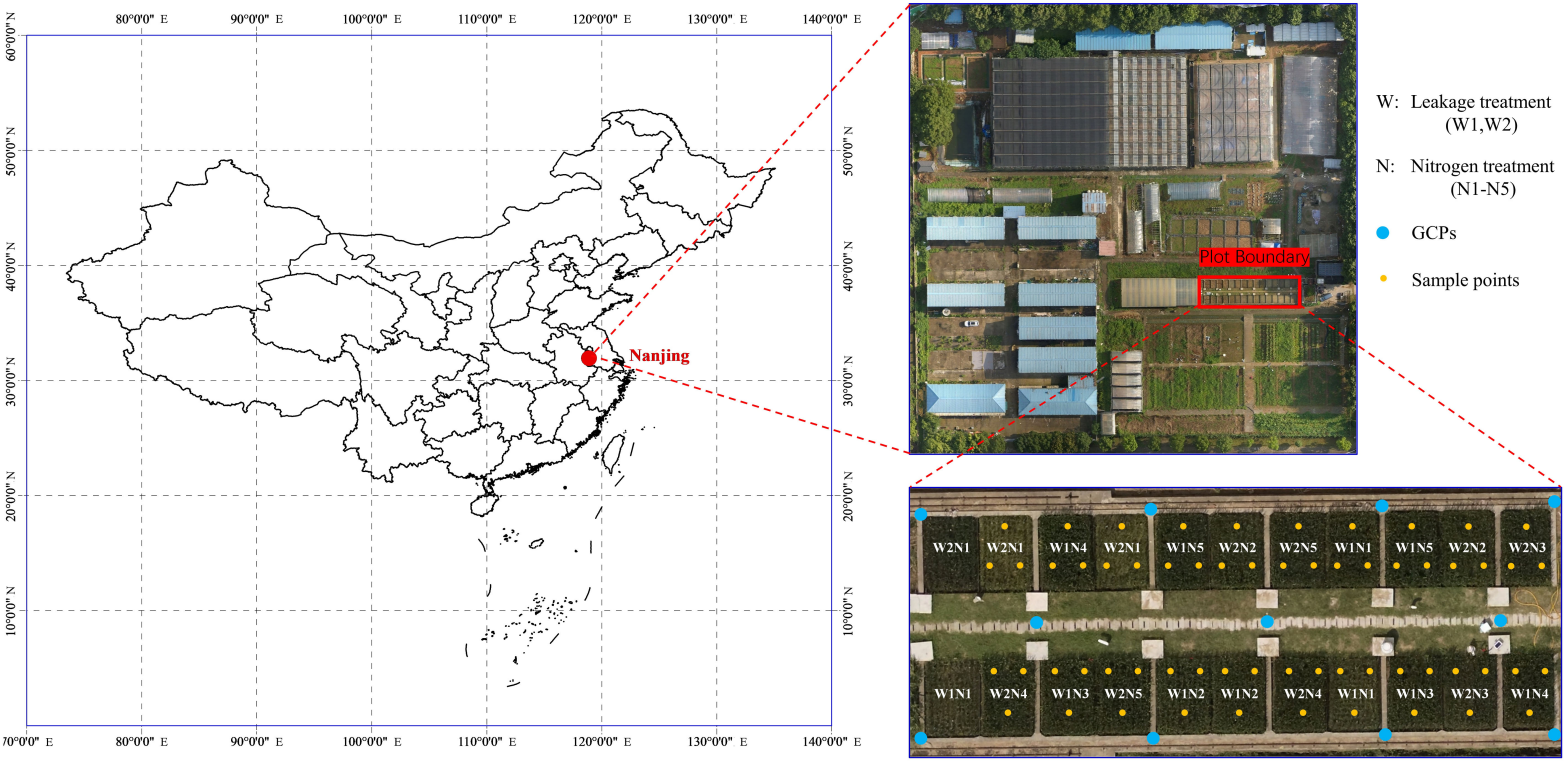
Study area and experiment treatments. W represents the leakage treatments (including W1: 3mm/day and W2: 5mm/day); N represents the nitrogen treatments (including N1-N5: 0, 150, 225, 300 and 375 kg/ha total pure nitrogen, respectively); GCP is abbreviation of gourd control points for geometric correction; Ground measurements in each sample point were averaged from 3 representative plants.

The experiment was conducted at the Jiangning Campus of Hohai University, Nanjing City, Jiangsu Province in China (31°54’57” N, 118°46’37” E). A total of 22 plots were set in this study, with a length × width of 2.5 m × 2.0 m and a depth of 2.0 m, which were cultivated with a rice–wheat rotation for many years. Rice cultivar (Nanjing-9108) was transplanted on 4 July with a spacing of 20 cm × 15 cm and harvested on 25 October 2021 under the controlled irrigation and drainage scheme. In order to obtain various spectral characteristics and photosynthetic characteristics parameters of rice canopy at different stages, five nitrogen fertilizer levels (N1–N5: 0, 150, 225, 300 and 375 kg/ha total pure nitrogen) and two infiltration levels (W1 and W2: 3 and 5 mm/day) were applied. The above two-factor complete experimental scheme was used for randomized design within the 22 plots. N fertilizers were employed as the base fertilizer (5 July), tiller initiation fertilizer (14 July), and spikelet-developing fertilizer (14 August) with the proportion of 40%, 30%, and 30% of total pure nitrogen, respectively. Phosphate (P) and potassium (K) fertilizers were applied once as the base fertilizer. All plots were well managed with practices commonly adopted by local farmers. The basic properties of the test soils are listed in **Table 1** (Chen et al., 2022a) and the location of the experimental area and the arrangement of experimental treatments are shown in **Figure 1**.

**Table 1:** Basic soil properties of different layers.

Soil layer (cm)	Soil particle fraction (%)			Bulk density (g cm^−3^)	Organic matter (%)	pH (H_2_O)
	Sand	Silt	Clay			
0–20	40.21 ± 9.06	38.22 ± 6.43	21.57 ± 3.26	1.36 ± 0.23	1.24 ± 0.06	6.94 ± 0.06
20–40	39.12 ± 6.31	39.16 ± 4.71	21.72 ± 2.63	1.40 ± 0.19	1.35 ± 0.06	6.97 ± 0.05
40–60	38.87 ± 5.46	39.86 ± 4.06	21.27 ± 2.83	1.43 ± 0.20	1.20 ± 0.08	6.85 ± 0.07
60–160	40.25 ± 5.02	38.12 ± 3.72	21.63 ± 2.41	1.48 ± 0.21	/	6.80 ± 0.04

### 2.2 UAV based multispectral data acquisition and processing

A DJI Innovation’s Phantom4-M (P4M) was employed as the phenotyping platform in this study. It is equipped with a multispectral camera with six CMOS, including one color sensor for visible light imaging (RGB) and five monochrome sensors for multispectral imaging. Each sensor has an effective pixel of 2.08 million, a lens field angle of 62.7°, and a focal length of 5.74 mm. Specific parameters of the sensor are shown in **Table 2**. The UAV-based multispectral image data were obtained under clear and cloudless weather conditions (10:00–14:00) at each rice growth stage. The UAV flew at an altitude of 15 m, with a heading overlap of 85% and a sideway overlap of 75%.

**Table 2:** Multispectral camera sensor parameters.

Band name	Abbreviations	Center wavelength (nm)	Band width (nm)	Resolution (pixels)
Blue	B	450	16	1,600 × 1,300
Green	G	560	16	1,600 × 1,300
Red	R	650	16	1,600 × 1,300
Red edge	RE	730	16	1,600 × 1,300
Near infrared	NIR	840	26	1,600 × 1,300

The multispectral original images of five bands acquired by each UAV flight sortie were exported into the PIE-UAV software (Piesat Information Technology Co., Ltd., China) to correct and splice into field orthophoto. The production steps of the orthophoto were as follows: (1) image matching: match the original images with 40,000 key and tie point limits by geographical location matching method; (2) image aligning: import ground control point (GCP) information and align the images with high adjustment accuracy, 0.05 pixel GCP measurement accuracy, and 0.5 pixel connection point matching accuracy; (3) DEM building: generate DEM data using a resolution of 1 GSD; (4) tessellation building: generate the tessellation line based on the Voronoi Geometry method; (5) orthophoto correction: correct the orthophoto with automatically calculated image resolution and the mosaic line mask method; (6) color balancing: set the number of pyramid layers to 3 for color homogenization of mosaic images; and (7) image mosaicking: resample the orthophoto by the cubic convolution method and export into Geo-Tiff format. The final size and resolution of the orthophoto were 9,012 × 5,126 pixels and 7.25 mm/pixel, respectively. ENVI 5.3 was used to perform layer stacking on Tiff images of each band to obtain five-band multispectral images, and the digital numbers (DNs) were transformed into reflectance by radiometric correction.

### 2.3 Field data collection

Simultaneous field measurements were conducted within the same day of the UAV multispectral image data acquisition, including rice PH, SPAD, and photosynthetic parameters Pn, Tr, and Gs. The PH values were measured with a soft ruler from the soil ground to the leaf tip (cm). The SPAD values were measured by the chlorophyll meter model (SPAD-502, Spectrum Technologies, Inc., NE, USA) and averaged from the measurements at the tip, middle, and base of each leaf. The photosynthetic parameters were measured by the portable photosynthesis system (LI-6800, LI-COR Inc., NE, USA) at 10:00–11:30 a.m. The measured leaf position was the middle of the latest fully unfolded leaf at the jointing–booting stage, and the middle of the panicle leaf at heading–flowering and ripening stages. Each parameter was averaged from three representative plants within a 30 cm × 30 cm quadrat, and three quadrats were measured for each plot. Thus, 60 groups of field data were obtained for each growth stage and 180 groups in the total growth season. The details of ground measurements and UAV flights are listed in **Table 3**.

**Table 3:** Ground and UAV data acquisition details.

Date	Growth stage	Temperature	Wind speed	Ground measurements	UAV data acquisition		
		(°C)	(m/s)		Time	Height (m)	Resolution (mm/pixel)
17 August 2021	Jointing–booting	30.38	1.90	PH, SPAD, Pn	10:45 a.m.	15.00	7.25
3 September 2021	Heading–flowering	31.08	4.30	PH, SPAD, Pn	11:10 a.m.	15.00	7.25
21 September 2021	Ripening	30.05	2.60	PH, SPAD, Pn	11:00 a.m.	15.00	7.25

It should be noted that the weather conditions in early September 2021 were mainly cloudy and rainy; the measurement on 3 September was the only relatively ideal condition. Therefore, the ground SPAD and Pn measurement at the heading–flowering stage and photography of UAV were affected to a certain extent.

### 2.4 Vegetation index and textural index calculation

#### 2.4.1 Vegetation index calculation

VI is established by the linear or nonlinear combination of different spectral band reflectances, which is a common method to retrieve physiological and biochemical indicators of crops (Zeng et al., 2022). A set of 25 commonly used VIs were employed in this study to investigate the relationship between VIs and rice photosynthetic parameters. Threshold processing was firstly performed on the stacked multispectral image to eliminate the influence of water on the reflectance. The canopy reflectance of each band within the 30 cm × 30 cm region of interest (ROI) was then averaged to calculate the VIs of each plot. The involved VIs and formulas are listed in **Table 4**.

**Table 4:** Vegetation indices and formula examined in this study.

Vegetation index	Abbreviations and formula
NIRv(Zeng et al., 2019)	$NIRv=b_{NIR}\frac{b_{NIR}-b_{R}}{b_{NIR}+b_{R}}$
Chlorophyll Index Green(Gitelson et al., 2005)	$CI_{green}=\frac{b_{NIR}}{b_{G}}-1$
Chlorophyll Index Red Edge (Gitelson et al., 2005)	$CI_{red\;edge}=\frac{b_{NIR}}{b_{RE}}-1$
Chlorophyll Vegetation Index (Gitelson et al., 2003)	$CVI=\frac{b_{NIR}\times b_{R}}{b_{G}{\;}^{2}}$
Difference Vegetation Index (Vincini et al., 2008)	*DVI=b*=*b*_*NIR*_−*b*_*R*_
Enhanced Vegetation Index (Jordan, 1969)	$EVI=2.5\frac{b_{NIR}-b_{R}}{b_{NIR}+6b_{R}-7.5b_{B}+1}$
Greenness Index (Huete et al., 2002)	$GI=\frac{b_{G}}{b_{R}}$
Green Normalized Difference Vegetation (Smith et al., 1995)	$GNDVI=\frac{b_{G}-b_{R}}{b_{G}+b_{R}}$
Modified Chlorophyll Absorption in Reflectance Index (Gitelson et al., 1996)	$MCARI=(b_{RE}-b_{R})-0.2\frac{\left(b_{RE}-b_{G}\right)}{b_{RE}/b_{R}}$
Modified Nonlinear Vegetation Index (Daughtry et al., 2000)	$MNVI=\frac{1.5(b_{NIR}{\;}^{2}-b_{R})}{b_{NIR}{\;}^{2}+b_{R}+0.5}$
Modified Soil Adjusted Vegetation Index (Gong et al., 2003)	$MSAVI=\frac{b_{NIR}+1-}{0.5\sqrt{{\left(2b_{NIR}+1\right)}^{2}-8(b_{NIR}-b_{R})}}$
Modified Simple Ratio (Goel and Qin, 1994)	$MSR=\frac{b_{NIR}/b_{R}-1}{\sqrt{b_{NIR}/b_{R}+1}}$
MERIS Terrestrial Chlorophyll Index (Chen, 1996)	$MTCI=\frac{b_{NIR}-b_{RE}}{b_{RE}-b_{R}}$
Modified Triangular Vegetation Index (Dash and Curran, 2004)	$MTVI=\frac{1.5(1.2(b_{NIR}-b_{G})-2.5(b_{R}-b_{G}))}{\sqrt{{\left(2b_{NIR}+1\right)}^{2}-(b_{NIR}-5\sqrt{b_{R}})}-0.5}$
Nonlinear Vegetation Index (Haboudane et al., 2004)	$NLI=\frac{b_{NIR}{\;}^{2}-b_{R}}{b_{NIR}{\;}^{2}+b_{R}}$
Normalized Difference Vegetation Index (Tucker et al., 1979)	$NDVI=\frac{b_{NIR}-b_{R}}{b_{NIR}+b_{R}}$
Optimization of Soil-Adjusted Vegetation Index (Rondeaux et al., 1996)	$OSAVI=\frac{1.16(b_{NIR}-b_{R})}{b_{NIR}+b_{R}+0.16}$
Renormalized Difference Vegetation Index (Roujean and Breon, 1995)	$RDVI=\frac{b_{NIR}-b_{R}}{\sqrt{b_{NIR}+b_{R}}}$
Ratio Vegetation Index 1 (Birth and McVey, 1968)	$RVI1=\frac{b_{NIR}}{b_{R}}$
Ratio Vegetation Index 2 (Xue et al., 2004)	$RVI12=\frac{b_{NIR}}{b_{G}}$
Structure Intensive Pigment Index (Blackburn, 1998)	$SIPI=\frac{b_{NIR}-b_{B}}{b_{NIR}+b_{B}}$
Transformed Chlorophyll Absorption in Reflectance Index (Haboudane et al., 2002)	$TCARI=3((b_{RE}-b_{R})-0.2(b_{RE}-b_{G})\frac{b_{RE}}{b_{R}})$
Triangular Vegetation Index (Broge and Leblanc, 2001)	*TVT*=60(*b*_*RE*_−*b*_*G*_)−100(*b*_*R*_−*b*_*G*_)
Visible Atmospherically Resistant Index (Gitelson et al., 2002)	$VARI=\frac{b_{G}-b_{R}}{b_{G}+b_{R}-b_{B}}$
Visible Difference Vegetation Index (Zhang et al., 2019)	$VDVI=\frac{2b_{G}-b_{R}-b_{B}}{2b_{G}+b_{R}+b_{B}}$

b_B_, b_G_, b_R_, b_RE_, and b_NIR_ represent blue (450 ± 16 nm), green (560 ± 16 nm), red (650 ± 16 nm), red edge (730 ± 16 nm), and near-infrared (840 ± 26 nm) band reflectance, respectively.

#### 2.4.2 Textural index calculation

Gray-level cooccurrence matrix (GLCM) (Haralick et al., 1973) was applied in this study to extract eight texture features from each band in the stacked image, including mean (MEAN), variance (VAR), homogeneity (HOM), contrast (CON), dissimilarity (DIS), entropy (ENT), second moment (SEC), and correlation (COR), and a total of 40 texture features (with a 3 × 3 pixel window size) were obtained. The same ROI size with VIs was used to extract texture features and the average value was taken. The extracted GLCM texture features were numbered in the order of MEAN, VAR, HOMO, CON, DIS, ENT, SEC, and COR of each band (in the order of band 1 to band 5) from 1 to 40. The normalized difference textural index (NDTI), difference textural index (DTI), and renormalized difference textural index (RDTI) were selected to construct TI involving two different texture features. The TI formulas are as follows:


$$\text{NDTI}=\frac{T_{1}-T_{2}}{T_{1}+T_{2}}$$

$$\text{DTI}=T_{1}-T_{2}$$

$\text{RDTI}=\frac{T_{1}-T_{2}}{\sqrt{T_{1}+T_{2}}}$ .

where *T*_1_ and *T*_2_ represented two random different texture features.

### 2.5 Modeling and validation

#### 2.5.1 Machine learning regression methods

Linear regression (LR), support vector regression (SVR), gradient boosting decision tree (GBDT), random forest (RF), and multilayer perceptron neural network (MLP) were employed in this study for Pn estimation. The *gridsearch* tuning results for the hyperparameters of each model are listed in **Table 5**, where the unmentioned hyperparameters were the default values.

**Table 5:** Tuned hyperparameters of models employed in this study.

Model	Tuned hyperparameters
SVR	kernel=‘rbf’; gamma= ‘auto’; C=1.0.
GBDT	n_estimators=100;max_features=‘none’;max_depth=‘adaptive’.
RF	n_estimators=100;max_features=‘none’;max_depth=‘adaptive’.
MLP	hidden_layer_sizes= (100),; activation=‘relu’; solver=‘lbfgs’; learning_rate=0.001.

LR, SVR, GBDT, RF, and MLP represent linear regression, support vector regression, gradient boosting decision tree, random forest, and multilayer perceptron neural network models, respectively.

(1) Linear regression: LR is a traditional algorithm based on classical statistics, which is the most commonly used model in the spectral inversion research because of its simple construction form and strong interpretation. Combined with the correlation analysis, the relationship between variables and target parameters can be directly reflected. In this study, the LR model with the ordinary least squares method was used for Pn multiple regression.

(2) Support vector regression: SVR is an important application branch of support vector machine (SVM), which seeks the optimal hyperplane by minimizing the total deviation of all sample points from the hyperplane (Cortes and Vapnik, 1995). Unlike ordinary least squares, the SVR model sets a threshold *ϵ* around the regression line such that all data points within *ϵ* are not penalized for their errors. Kernel function, gamma, and C are crucial parameters in the SVR model and have been tuned through the *gridsearch* method in the *sklearn* package.

(3) Gradient boosting decision tree: GBDT is an iterative decision tree algorithm with a “boosting” ensemble learning method (Friedman, 2001; Wu et al., 2020). The basic learners [usually classification and regression tree (CART)] in the GBDT model have strong dependencies between each other and are trained by progressive iterations based on the residuals. The results of all basic learners are added together as the final output, which grant GBDT great advantages in overfitting and computational cost fields and reduce bias at the same time.

(4) Random forest: RF is one of the most popular tree algorithms proposed by Breiman (2001) based on the bagging idea of ensemble learning. RF applies the “bootstrap” method to retrieve samples to train the N basic learners (usually CART) in parallel without dependence. The final output of the RF model is derived by combining results of the basic models with the “voting” method, which makes the RF model insensitive to outlier variable values.

(5) Multilayer perceptron neural network: MLP is generally composed of a fully connected input layer, a hidden layer, and an output layer, in which the hidden layer can be multiple (Khoshhal and Mokarram, 2012). As the most basic form of feed-forward neural network, the MLP model has been widely applied in the analysis of various complex problems and is also the foundation of CNN, deep neural network (DNN), and other complex neural networks. A typical three-layer MLP model was used in this study and parameters were well tuned.

#### 2.5.2 Model validation and evaluation

Sixty groups of multispectral data and field measured data in each growth stage were divided into training and validation sets by the 10-fold cross-validation method. Each time, 90% and 10% of the data were employed as training and validation sets, respectively; this process continues 10 times until all the samples have been predicted once and only once. The model final performance was averaged from the evaluation criteria in the cross-validation. To comprehensively evaluate the model performance of Pn estimation, the mean square error (MSE), mean absolute error (MAE), explained variance score (EVS), and coefficient of determination (*R* ^2^) were considered in this study as the evaluation criteria. All model code and evaluation criteria calculations were written in Python3.2 and implemented in a laptop with Intel Core i7-9750H CPU @2.60 GHz, NVDIA GeForce GTX 1660 Ti GPU, and 16 GB of RAM.


$$MSE=\frac{1}{n}\sum_{i=1}^{n}{\left(y_{i}-{\hat{y}}_{i}\right)}^{2}$$

$$MAE=\frac{1}{n}\sum_{i=1}^{n}\left|y_{i}-{\hat{y}}_{i}\right|$$

$$EVS=1-\frac{VAR\left(y_{i}-{\hat{y}}_{i}\right)}{VAR\left(y_{i}\right)}$$

$$R^{2}=\frac{\sum_{i=1}^{n}{\left({\hat{y}}_{i}-\bar{y}\right)}^{2}}{\sum_{i=1}^{n}{\left(y_{i}-\bar{y}\right)}^{2}}$$

where *y*_*i*_ , ${\hat{y}}_{i}$ and $\bar{y}$ epresent the measured value, the mean measured value, and the estimated value, respectively. *n* represents the number of the results. VAR represents the variance of the results. MSE and MAE are in the same unit with the measured value, ranging from 0 (optimum value) to +*∞* (worst value). EVS and *R* ^2^ are dimensionless, ranging from 0 (worst value) to 1 (optimum value).

## 3 Results

### 3.1 Rice photosynthetic traits and canopy multispectral feature response to different treatments

#### 3.1.1 Rice plant height, SPAD, and net photosynthetic rate

**Figure 2:**
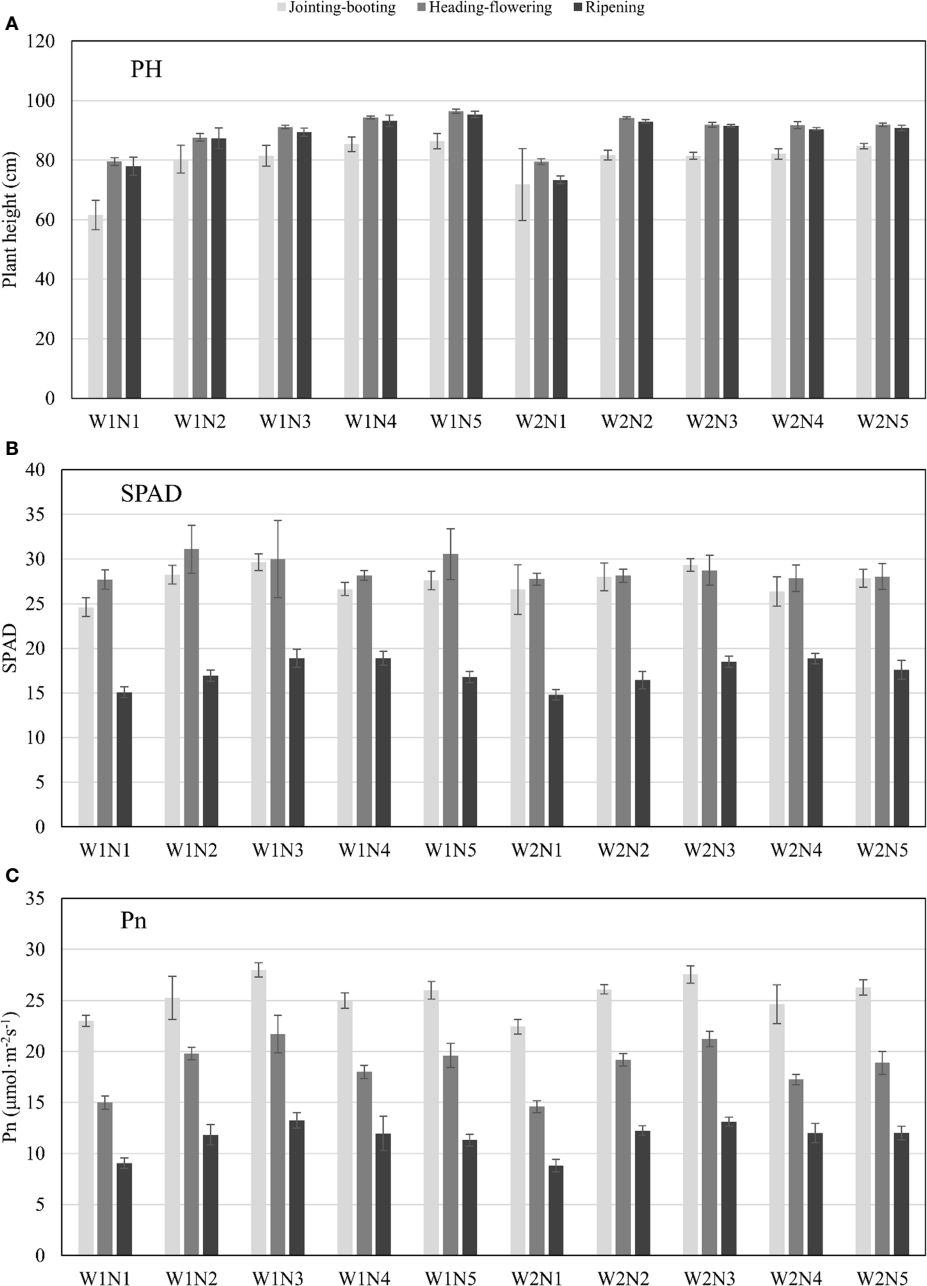
PH, SPAD and Pn of rice response to different treatments at different stages. **(A)** PH represents plant height (cm); **(B)** SPAD is relative chlorophyll content; **(C)** Pn represents the net photosynthetic rate (umol m-2s-1). W represents the leakage treatments (including W1: 3mm/day and W2: 5mm/day); N represents the nitrogen treatments (including N1-N5: 0, 75, 150, 225 and 300 kg/ha total pure nitrogen, respectively). The above ground measurements were conducted at the same time with UAV flight at 3 growth stages.

The PH, SPAD, and Pn of tested rice at the jointing–booting, heading–flowering, and ripening stage are shown in **Figure 2**, respectively. PH increased obviously with the increase of nitrogen (N) application and advancement of growth stage, while it decreased slightly with ear filling at the ripening stage (**Figure 2A**). When PH reached the highest at the heading–flowering stage, the average rice PH with N2–N5 level under W1 (low leakage) treatment was 10.11%, 14.51%, 18.53%, and 21.23% higher than that under the N1 level, respectively, and 18.48%, 15.59%, 15.47%, and 15.59% higher than the N1 level, respectively, for W2 treatment. PH under W2 (high leakage) treatment was significantly higher than those under W1 treatment at N1 and N2 levels in the jointing–booting and heading–flowering stage, respectively, but the difference was not obvious under other N applications.

As shown in **Figure 2B**, SPAD generally showed a trend of initially increasing (N1–N3) then decreasing (N3–N4) and finally ending up with a small increase (N4–N5) with the increase of nitrogen application under the same leakage conditions. The maximum value of SPAD in each growth stage almost appeared at the N3 level, while the lowest value was found at the N1 level without N application. The SPAD value reached the maximum at the heading–flowering stage, and the average SPAD values of N2–N5 levels were 12.27%, 8.30%, 1.62%, and 10.29% higher than the N1 level for W1 leakage treatment and 1.44%, 3.60%, 0.36%, and 1.08% higher than the N1 level for W2 leakage treatment, respectively. For the same N application level, W1 leakage treatment could increase the SPAD value of N1–N5 levels by −0.18%, 10.48%, 4.35%, 1.08%, and 8.91%, respectively, compared with W2.

It can be seen from **Figure 2C** that the Pn of rice decreased with the advancement of the rice growth stage. Pn under different N treatments showed a generally similar change trend with SPAD, increasing with the increase of N application at N1 to N3 levels and reaching the maximum at N3, decreasing at N4, and reverting at the N5 level [the Pn increase from N4 to N5 is not significant (*p* > 0.05)]. At the jointing–booting stage when photosynthesis was most vigorous, the average Pn values under N2–N5 levels with W1 treatment were 9.78%, 21.74%, 8.69%, and 13.04% higher than the N1 level, respectively, and 16.36%, 22.87%, 9.72%, and 17.16% higher than the N1 level under the conditions of W2 treatment, respectively, which indicated that excessive application of N fertilizer might inhibit photosynthesis. Under the same N application level, the Pn with W1 treatment was slightly higher than that with W2 treatment, indicating that low leakage intensity could promote leaf photosynthesis to a certain extent.

#### 3.1.2 Rice canopy multispectral reflectance characteristics

**Figure 3:**
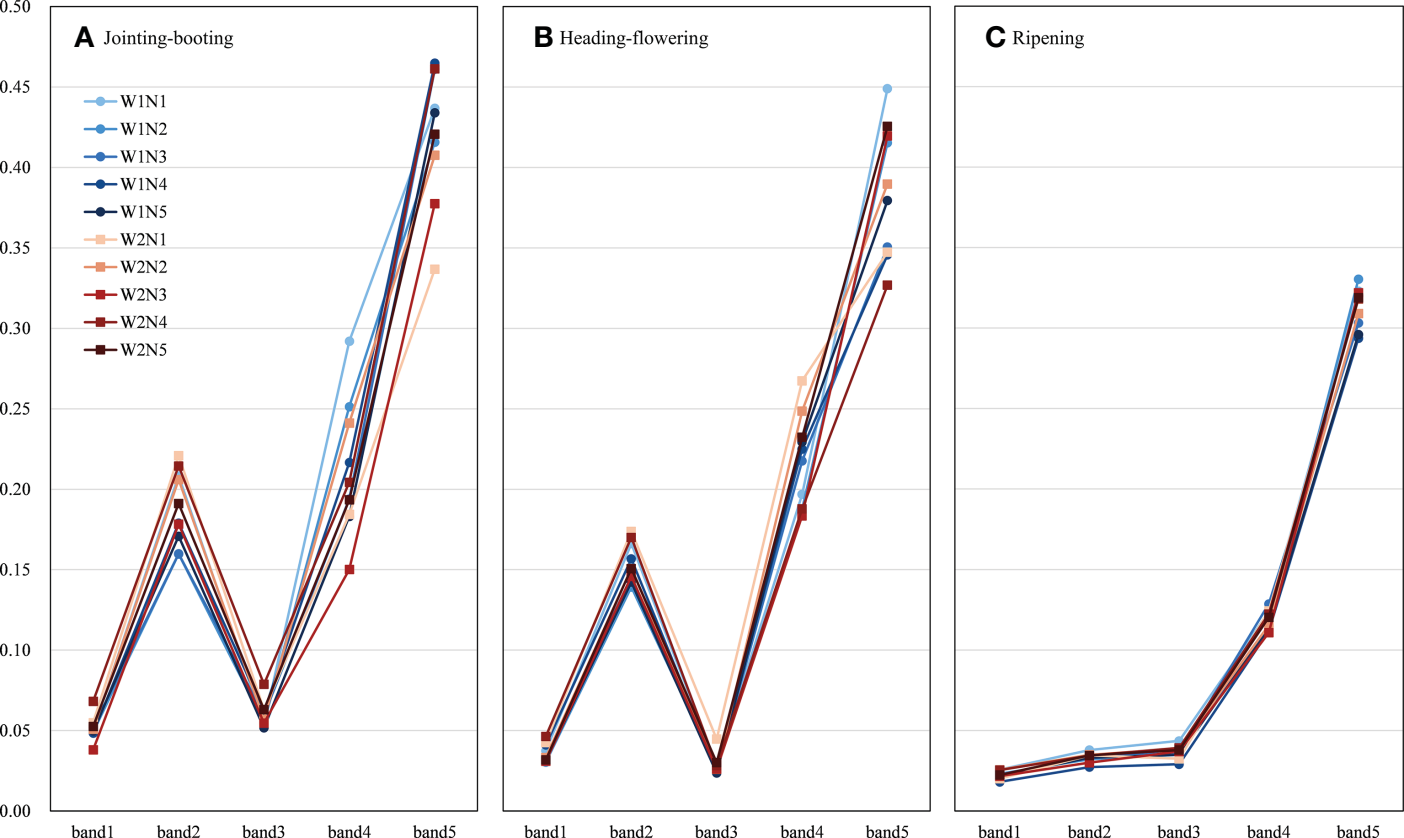
Average value of canopy band reflectance response to different treatments at different stages. **(A-C)** are the reflectance at jointing-booting, heading-flowering and ripening stage, respectively. Band1-band5 represent blue (450±16 nm), green (560±16 nm), red (650±16 nm), red edge (730±16 nm), near-infrared (840±26 nm) reflectance, respectively. W represents the leakage treatments (including W1: 3mm/day and W2: 5mm/day); N represents the nitrogen treatments (including N1-N5: 0, 75, 150, 225 and 300 kg/ha total pure nitrogen, respectively).

**Figure 3** illustrates the rice canopy multispectral reflectance of blue (band 1), green (band 2), red (band 3), red edge (band 4), and near infrared (band 5) with different treatments at three growth stages. In general, the average reflectance value of all five bands decreased as the growth stage progressed. As one of the most representative features in the crop spectral curve, the band 5 reflectance value ranged from 0.337 to 0.465 at the jointing–booting stage, while it decreased slightly to 0.327–0.449 at the heading–flowering stage, and finally dropped to approximately 0.030 at the ripening stage. Band 2 was the most intuitive band visible to the naked eye that could represent the nutrient statue and growth stage of crops, which reached a maximum of 0.160–0.221 at the jointing–booting stage, decreased to 0.139–0.174 with heading and flowering, and finally decreased to 0.027–0.038 with the yellowing of leaves and ears at the ripening stage.

For the same N application level at the jointing–booting stage, the average reflectance values of band 1, band 2, and band 3 under the W1 leakage treatment were generally lower than those under the W2 leakage treatment, except that W1N3 had higher band 1 and band 3 values than W2N3. However, the average reflectance values of band 4 and band 5 showed opposite trends; specifically, W1 leakage treatment could increase the reflectance of band 4 and band 5 compared with W2 treatment and the N3 level improved the most. It could also be found from **Figure 3A** that lower leakage treatment had a steeper increase from band 3 to band 4, which indicated the better growth status. Under the same leakage treatment, the average reflectance values of band 1 to band 4 generally presented a trend of initially decreasing (N1–N3), then increasing (N3–N4), and finally ending up with a small decrease (N4–N5), while the reflectance value of band 5 increased with the N application level, but the law was not obvious. Moreover, the average reflectance value of these five bands did not show a certain rule in the heading–flowering stage and the ripening stage, which might be due to the influence of rice heading and flowering, here leaves and ear yellowing in the growth process on the multispectral characteristics of the canopy.

### 3.2 Correlation analysis between VIs, TIs, and Pn

Pearson’s correlation coefficients (*r* values) between the above 25 VIs and Pn at three growth stages are listed in **Table 6**. The code for *r* value calculation and significance analysis was written in Python3.2 with the *scipy* package (1.20.3). Generally, the VIs had a better relationship with Pn at the jointing–booting stage, but the *r* value became worse as the growth stage advanced; however, it might be that the number of samples was relatively small and therefore no VIs passed the highly significant correlation test (*p*< 0.01). To be specific, CI_green_, CVI, MNVI, NLI, OSAVI, RDVI, and RVI2 achieved a significant positive correlation (*p*< 0.01) with Pn at the jointing-boosting stage, with *r* value ranging from 0.3330 to 0.3893. NIRv, DVI, EVI, MSAVI, MSR, NDVI, RVI1, SIPI, and TCARI also had a satisfactory *r* value (*p*< 0.05) with absolute value between 0.2753 and 0.3262. At the heading–flowering stage, only CI_green_, RVI2, and SIPI showed a significant relationship and NDVI, NLI, TCARI, and VDVI had a relatively higher *r* value. When the crop proceeded to the ripening stage, no VIs could achieve a satisfactory *r* value with Pn. The VIs employed for Pn estimation were thus selected based on the *r* value at different stages, and the selected VIs are shown in bold in **Table 6**.

**Table 6:** Correlation coefficient (*r* value) between selected VIs and Pn.

VIs	Jointing–booting (*n* = 60)	Heading–flowering (*n* = 60)	Ripening (*n* = 60)
NIRv	0.3262*	0.2034	−0.0903
CI*_green_*	0.3893**	0.3490**	**0.1693**
CI*_red edge_*	0.1938	0.1046	0.0649
CVI	0.3396**	0.1155	**0.1605**
DVI	0.3186*	0.1737	−0.1083
EVI	0.2803*	0.1333	−0.0949
GI	−0.1549	−0.0196	0.0137
GNDVI	−0.1473	0.1699	−0.0531
MCARI	−0.0091	−0.0642	−0.095
MNVI	0.3342**	0.2119	−0.0798
MSAVI	0.3281*	0.2194	−0.0820
MSR	0.3157*	0.1905	0.0926
MTCI	0.1369	0.0886	0.0742
MTVI	0.1419	0.1113	−0.0846
NDVI	0.3178*	0.3166*	0.0370
NLI	0.3386**	0.2661*	−0.0056
OSAVI	0.3330**	0.2622	−0.0487
RDVI	0.3342**	0.2259	−0.0746
RVI1	0.3136*	0.1038	0.1223
RVI2	0.3893**	0.3490**	**0.1693**
SIPI	0.3258*	0.3381**	0.0648
TCARI	−0.2753*	−0.3014*	**−0.2131**
TVI	−0.1262	−0.2094	**−0.1258**
VARI	−0.0984	0.0632	−0.0685
VDVI	−0.1881	0.2746*	−0.0680

* and ** represent significant level p< 0.05 and p< 0.01, respectively. The number is the sample size of the data. VIs corresponding to the bolded value were selected as the inputs for machine learning modeling at different growth stages.

**Figure 4:**
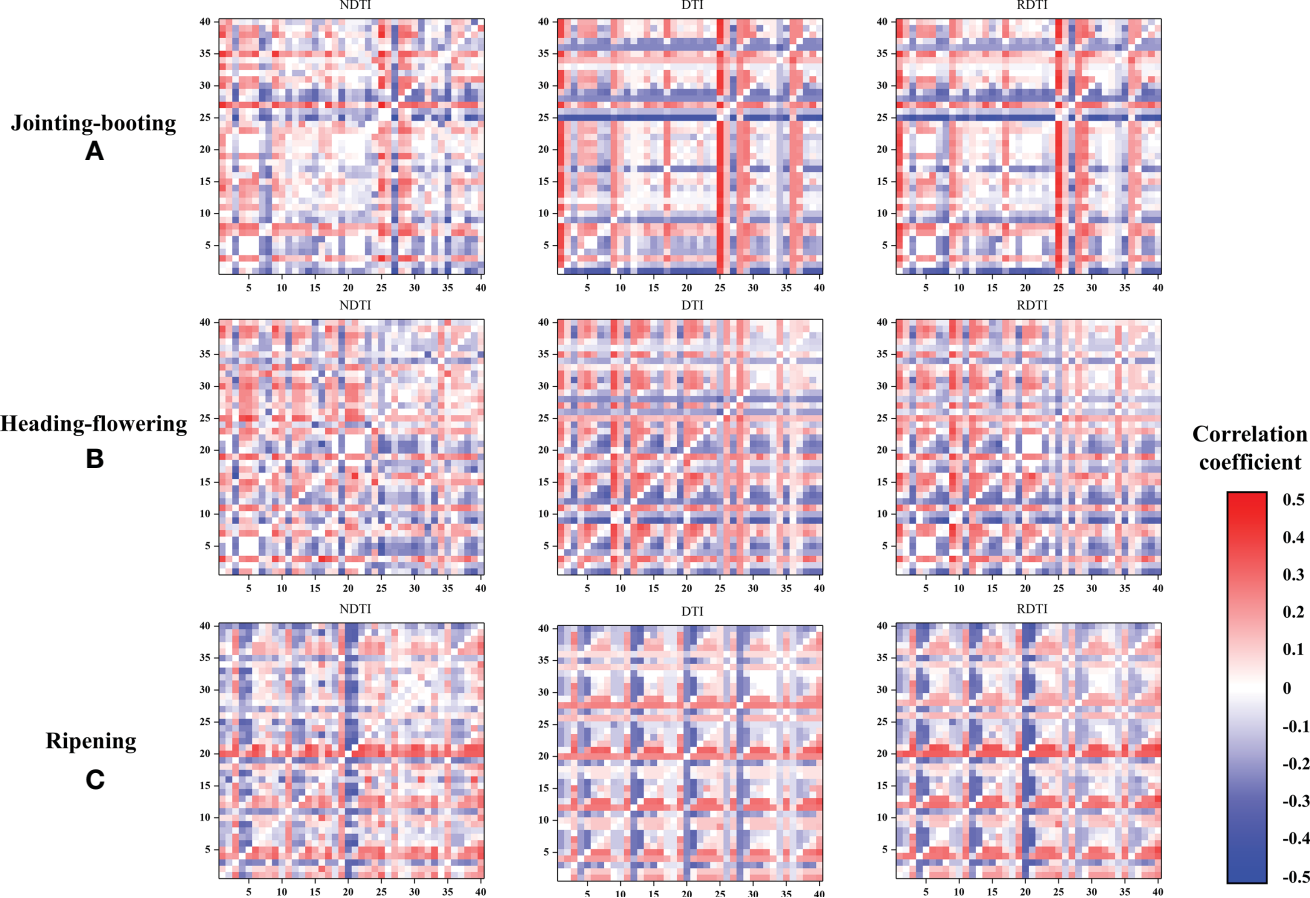
Correlation coefficient between Pn and TIs with different textural features combination. **(A-C)** are the correlation coefficient values at jointing-booting, heading-flowering and ripening stage, respectively. NDTI, DTI, RDTI represent normalized difference textural index, difference textural index and renormalized difference textural index, respectively. X-axis and Y-axis legends are the texture features in order of NO.1-40. The coloration in the thermal map is based on the correlation (r value) between TIs and Pn.

The NDTI, DTI, and RDTI were calculated using any two texture features from NO. 1 to NO. 40 and the correlation thermal map in **Figure 4** was thus drawn according to the *r* value between TIs and Pn. Because the order of the two selected texture features was different, the correlation *r* value in the figure presented positive and negative axis symmetry. Taking the thermal map at the jointing–booting stage as an example, it could be found that there were obviously deeper red and blue lines in the DTI and RDTI figure, indicating that the DTI and RDTI composed of any of the NO. 1 (MEAN1) and NO. 25 (MEAN4) features with the other feature had a better relationship with Pn (*r* value mainly approximately 0.35 and 0.39, respectively). Scattered hot spots with high *r* value could be seen in the RDTI figure, but no dominant texture feature could be found. For each TI, the feature combination with the highest correlation with Pn was selected and the results at different growth stages are listed in **Table 7**.

**Table 7:** Compositions of selected TIs and its correlation with Pn.

TIs/stages	Jointing–booting		Heading–flowering		Ripening	
	Combination	*r*	Combination	*r*	Combination	*r*
NDTI	MEAN4, HOMO4	0.4361	COR2, HOMO1	0.4052	ENT2, DIS3	0.3526
DTI	MEAN4, DIS2	0.4042	MEAN2, COR1	0.3650	COR5, DIS3	0.3348
RDTI	MEAN4, COR5	0.4169	MEAN2, COR1	0.4016	COR5, DIS3	0.3808

NDTI, DTI, and RDTI represent normalized difference textural index, difference textural index, and renormalized difference textural index, respectively. The texture features mean, variance, homogeneity, contrast, dissimilarity, entropy, second moment, and correlation extracted by the GLCM method are abbreviated as MEAN, VAR, HOM, CON, DIS, ENT, SEC, and COR, respectively. The number after the abbreviation represents the band where the feature is extracted.

### 3.3 Estimation rice Pn from VIs at different growth stages

**Figure 5:**
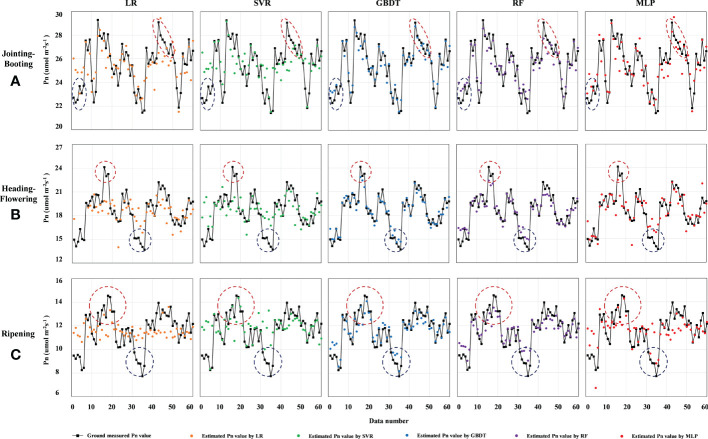
Comparison of estimated and measured Pn values of different models at certain stages. **(A-C)** are the comparison of estimated and measured Pn values at jointing-booting, heading-flowering and ripening stage, respectively. LR, SVR, GBDT, RF and MLP represent linear regression, support vector regression, gradient boosting decision tree, random forest and multilayer perceptron neural networks model, respectively. Pn represents the net photosynthetic rate (umol m-2s-1). The dashed blue and red circle in each figure are used to compare the fitting between the estimated and measured value when Pn value is low and high, respectively.

The accuracy comparison results between LR, SVR, GBDT, RF, and MLP models based on the selected VIs are listed in **Table 8** and all criteria indices were calculated by the average of 10-fold cross-validation results. At the jointing–booting stage, most VIs showed good correlation with Pn and a total of 16 VIs were selected for modeling; therefore, the models achieved the relatively highest accuracy compared with those at other growth stages. Specifically, GBDT achieved the highest average accuracy (with an MSE of 0.253 μmol m^−2^ s^−1^, an MAE of 0.414 μmol m^−2^ s^−1^, an EVS of 0.938, and an *R* ^2^ of 0.938), while SVR models attained the worst performance (with an MSE of 2.512 μmol m^−2^ s^−1^, an MAE of 1.155 μmol m^−2^ s^−1^, an EVS of 0.390, and an *R* ^2^ of 0.383). RF, MLP, and LR models ranked second, third, and fourth, respectively. **Figure 5A** also shows that the Pn estimated value of GBDT was the closest to the measured Pn value in each validation set. The Pn estimated values of LR and SVR models were almost concentrated in the range of 24–27 μmol m^−2^ s^−1^; thus, the estimation accuracy was not satisfactorily compared with GBDT, RF, and MLP models, where the Pn values were relatively lower and higher (inside the blue and red dashed circle). The correlation between VIs and Pn gradually weakened and inputted VIs thus decreased in number as the growth stage progressed; therefore, the model estimation accuracy decreased to a certain extent without ranking change. In detail, the LR model suffered the biggest loss in estimation accuracy with an *R* ^2^ value decreasing to 0.296 at the heading–flowering stage and then to 0.125 at the ripening stage. However, GBDT and RF models still showed good performance with *R* ^2^ values of 0.928 and 0.869 at the heading–flowering stage and 0.863 and 0.815 at the ripening stage, respectively. **Figures 5B, C** also demonstrated that the GBDT and RF models could better describe the relationship between VIs and Pn in the value full range at different growth stages. Although the performance of the MLP model ranked third, the performance was not ideal when compared with GBDT and RF models for both lower and higher PN value estimation. In conclusion, the accuracy of modes for estimating Pn value at the jointing–booting stage was relatively best and the GBDT model could be highly recommended for Pn estimation during the rice whole growth season.

**Table 8:** Model performance for Pn estimation based on VIs.

Growth stage	Model	MSE (μmol m^−2^ s^−1^)	MAE (μmol m^−2^ s^−1^)	EVS	*R* ^2^
Jointing–booting	LR	1.859	1.128	0.543	0.543
	SVR	2.512	1.155	0.390	0.383
	GBDT	0.253	0.414	0.938	0.938
	RF	0.521	0.604	0.872	0.872
	MLP	0.942	0.777	0.768	0.768
Heading–flowering	LR	4.165	1.612	0.298	0.296
	SVR	4.388	1.589	0.261	0.258
	GBDT	0.425	0.505	0.928	0.928
	RF	0.774	0.691	0.869	0.869
	MLP	1.948	1.06	0.671	0.671
Ripening	LR	2.413	1.296	0.125	0.125
	SVR	2.293	1.061	0.205	0.169
	GBDT	0.377	0.507	0.863	0.863
	RF	0.511	0.610	0.815	0.815
	MLP	1.490	0.930	0.460	0.460

LR, SVR, GBDT, RF, and MLP represent linear regression, support vector regression, gradient boosting decision tree, random forest, and multilayer perceptron neural network models, respectively.

### 3.4 Estimation rice Pn from fused VIs, TIs, and basal growth index

In order to further improve the Pn estimation accuracy, TIs (NDTI, DTI, and RDTI) and basal growth index (PH and SPAD) were introduced based on the input VIs. The accuracy comparison result of different models under different input combinations is shown in **Table 9**. After adding the TIs inputs for Pn estimation, all models performed higher accuracy at the jointing–booting stage with the MSE of LR, SVR, GBDT, RF, and MLP decreasing by 0.090, 0.370, 0.126, 0.113, and 0.057 μmol m^−2^ s^−1^, respectively, and the *R* ^2^ increasing by 0.022, 0.090, 0.031, 0.028, and 0.015, respectively. As the basal growth index further increased, the final *R* ^2^ of the employed models increased to 0.792, 0.565, 0.987, 0.943, and 0.822, respectively. It is possible that the basal growth index improved the model accuracy slightly more than TIs because the difference in PH could reflect the stress of crops to a certain extent, and SPAD is directly related to chlorophyll content, which directly affects photosynthesis. The same improvement effect of model accuracy could also be found at the heading–flowering and ripening stages, and although the improvement of GBDT and RF models was relatively small (with *R* ^2^ increasing to 0.062 and 0.031 for VIs + TIs input and 0.113 and 0.132 for VIS + TIs + PH and SPAD input), they were still the top two models among the employed models. The greatest improvement could be found in the LR model, with an *R* ^2^ increase of 0.139 and 0.470 at the heading–flowering stage and 0.114 and 0.471 at the ripening stage under the VIs + TIs and VIS + TIs + PH and SPAD input combination, respectively. The accuracy of the SVR model also had been improved greatly, but it still is the lowest among the five models. In conclusion, both TIs and the basal growth index could obviously improve the model accuracy for Pn estimation and the PH and SPAD had a better effect compared with the TIs in this study, which significantly improved the model performance at the heading–flowering and ripening stages, especially for LR and MLP models.

**Table 9:** Model performance for Pn estimation based on different input combination.

Growth stage	Model	VIs		VIs + TIs		VIs + TIs + PH and SPAD	
		MSE	*R* ^2^	MSE	*R* ^2^	MSE	*R* ^2^
		(μmol m^−2^ s^−1^)		(μmol m^−2^ s^−1^)		(μmol m^−2^ s^−1^)	
Jointing–booting	LR	1.859	0.543	1.769	0.565	0.846	0.792
	SVR	2.512	0.383	2.142	0.473	1.770	0.565
	GBDT	0.253	0.938	0.127	0.969	0.053	0.987
	RF	0.521	0.872	0.408	0.900	0.232	0.943
	MLP	0.942	0.768	0.885	0.783	0.723	0.822
Heading–flowering	LR	4.165	0.296	3.345	0.435	1.384	0.766
	SVR	4.388	0.258	4.052	0.315	3.471	0.413
	GBDT	0.425	0.928	0.321	0.946	0.052	0.991
	RF	0.774	0.869	0.768	0.870	0.174	0.971
	MLP	1.948	0.671	1.619	0.726	1.147	0.806
Ripening	LR	2.413	0.125	2.100	0.239	1.116	0.596
	SVR	2.293	0.169	2.229	0.192	1.834	0.335
	GBDT	0.377	0.863	0.207	0.925	0.066	0.976
	RF	0.511	0.815	0.426	0.846	0.146	0.947
	MLP	1.490	0.460	1.290	0.532	0.973	0.647

LR, SVR, GBDT, RF, and MLP represent linear regression, support vector regression, gradient boosting decision tree, random forest, and multilayer perceptron neural network models, respectively. VIs and TIs represent vegetation indices and textural indices, respectively. PH represents plant height (cm) and SPAD is relative chlorophyll content.

## 4 Discussion

### 4.1 Relationship between rice growth and canopy multispectral feature

The ground sample results showed that the SPAD and Pn generally increased with nitrogen application (N1–N3 levels), then decreased at the N4 level, and finally recovered at the N5 level under the same leakage treatment at the jointing–booting stage, which also indicated that proper N application could improve the photosynthesis, while excessive N application not only had a poor effect on photosynthesis, but also affected plant growth and increased risk of contamination during leakage and drainage. This phenomenon was similar to that found by Cisse et al. (2020). It might due to the fact that there was no significant difference in Rubisco activity and non-photochemical quenching (NPQ) between the N4–N5 and the N3 level; thus, excessive energy could not be dissipated by NPQ, leading to oxidative stress, resulting in a decrease in Pn when excessive nitrogen was applied. An opposite trend could be found for canopy multispectral reflectance as the N application increased. Band 1 to band 4 generally decreased when N application increased from the N1–N3 level, while they slightly increased at the N4 level then decreased again at the N5 level; however, band 5 reflectance consistently increased with N level, which was consistent with previous studies on other crops Qiu et al. (2015). Generally, crops with good growth have a lower reflectance and a steeper increase from the red to the NIR band; thus, the variation trend of the canopy reflectance was consistent with SPAD and PN, which also provides a theoretical basis for the inversion of photosynthetic characteristic parameters using VIs.

### 4.2 Limitations and suggestions on Pn estimation using VIs and TIs in this study

Based on the result and analysis in *Section 3.1.1*, the relationship between VIs and Pn decreased with the growth stage, which might be due to the influence of heading and flowering, although panicle photosynthesis is also an important part of crop canopy photosynthesis and contributes significantly to grain formation. However, due to the limitation that the photosynthetic measurement equipment used in this paper could only be used to measure leaves, the canopy photosynthesis was thus approximately the photosynthetic capacity of the included leaves. Therefore, after rice heading and flowering, the spectral reflectance of the canopy was affected to a certain extent and the correlation based on the above data decreased significantly. In order to improve the estimation accuracy of Pn at the heading–flowering stage, the image segmentation should be carried out first to remove the panicle reflectance image. Meanwhile, the method of canopy photosynthesis measurement and the effect of panicle on photosynthetic contribution and reflectance should be revised and improved in future studies.

According to the correlation analysis results of TIs during the whole growth season in **Figure 4**, it could be concluded that most of the dominant texture features were extracted from band 1 to band 3, while the TIs constructed by the features extracted from band 4 and band 5 were not that satisfactory. Specifically, MEAN and COR texture features were more included in the optimal features for NDTI, DTI, and RDTI construction, because MEAN represents the average of moving windows containing targets and backgrounds, which can smooth the image and reduce the interference of background factors, while COR can reflect the local gray-level correlation in the image and distinguish the differences of image texture in all directions. In addition, window sizes of 6 × 6, 9 × 9, and 12 × 12 were also employed in the GLCM for textural feature extraction; however, the performance was not much different from that of the 3 × 3 window size, which might be due to the high resolution (4.5 mm/pixel) of each pixel in the image. Therefore, improving the resolution of visible images to extract higher-quality TIs might be an economical and practical approach to improve the accuracy of Pn estimation.

### 4.3 Influence of input combination on Pn estimation performance

The model performance under the different input combinations of VIs, VIs + TIs, and VIs + TIs + PH and SPAD concludes that the fusion of VIs and TIs could effectively improve the accuracy of Pn estimation because the VIs contain the canopy reflectance features and are more sensitive to the nutrition variations, while the TIs could better reflect the difference in canopy structure. The results were also consistent with previous studies (Liu et al., 2019; Zhang et al., 2021) in that this fusion combination could improve the estimation accuracy of biomass, LAI, nitrogen nutrition, and potassium nutrition and accumulation. Furthermore, the addition of PH and SPAD brought higher accuracy, which could attribute its success to the high correlation between PH and N nutrition status, SPAD and chlorophyll content, brought by the obvious difference of 5 N treatment. To sum up, more different kinds of data could introduce more direct or indirect related features, and it is also suggested that stacking and blending ensemble learning methods (Wu et al., 2021) could be employed to combine the model ability of feature extraction and analysis based on different principles in future research to improve the model accuracy for Pn estimation, which is also the purpose and significance of developing agricultural big data and agricultural intelligent models.

## 5 Conclusion

This paper studied and revealed the responses of canopy multispectral band reflectance and rice net photosynthetic rate (Pn) to different nitrogen applications and leakage treatments through different growth stages under controlled irrigation and drainage schemes. The relationship between VIs, TIs, and Pn based on the UAV multispectral image was comprehensively analyzed and focused on. The performance of LR, SVR, GBDT, RF, and MLP models for Pn estimation under different input combinations was evaluated and compared at the jointing–booting, heading–flowering and ripening stages. The results indicated that the selected VIs and TIs had a relatively better correlation relationship with Pn at the jointing–booting stage, while only a moderate correlation at the heading–flowering stage and an unsatisfactory correlation at the ripening stage could be found. Therefore, the employed models generally had a better performance during the jointing–booting stage and the accuracy decreased as the growth stage progressed. Among the five used models, GBDT and RF models achieved the highest and most stable accuracy in the whole growth season and could be highly recommended for Pn estimation in the paddy field. Meanwhile, the fusion of VIs with TIs and basal growth index could significantly improve the model accuracy, and the plant height (PH) and SPAD had a better effect on performance improvement compared with NDTI, DTI, and RDTI employed in this study. The techniques and results presented in this paper could be valuable for rice field-scale photosynthetic monitoring, which could assist further stress detection and yield prediction.

## Data availability statement

The raw data supporting the conclusions of this article will be made available by the authors, without undue reservation.

## Author contributions

Conceptualization, TW and WZ. Methodology, TW and MC. Software, TW. Validation, TW, ZW and MC. Formal analysis, SW. Data curation, LQ. Writing—original draft preparation, TW. Writing—review and editing, TW, and LQ. Visualization, SW. Supervision, GS and XJ. Project administration, XJ. Funding acquisition, XJ. All authors contributed to the article and approved the submitted version.
