# Corrigendum: Retrieving rice (*Oryza sativa* L.) net photosynthetic rate from UAV multispectral images based on machine learning methods

**DOI:** 10.3389/fpls.2023.1229908

**Published:** 2023-06-13

**Authors:** Tianao Wu, Wei Zhang, Shuyu Wu, Minghan Cheng, Lushang Qi, Guangcheng Shao, Xiyun Jiao

**Affiliations:** ^1^ College of Agricultural Science and Engineering, Hohai University, Nanjing, China; ^2^ State Key Laboratory of Hydrology-Water Resources and Hydraulic Engineering, Hohai University, Nanjing, China; ^3^ Cooperative Innovation Center for Water Safety and Hydro Science, Hohai University, Nanjing, China; ^4^ Jiangsu Key Laboratory of Crop Genetics and Physiology/Jiangsu Key Laboratory of Crop Cultivation and Physiology, Agricultural College, Yangzhou University, Yangzhou, China

**Keywords:** UAV multispectral remote sensing, rice canopy, net photosynthetic rate, vegetation index, textural index, machine learning

In the published article, there was an error in the caption for **Figure 1** as published. The explanation of N was displayed as “N represents the nitrogen treatments (including N1-N5: 0, 75, 150, 225 and 300 kg/ha total pure nitrogen, respectively”. The corrected **Figure 1** caption appears below:

**Figure 1:**
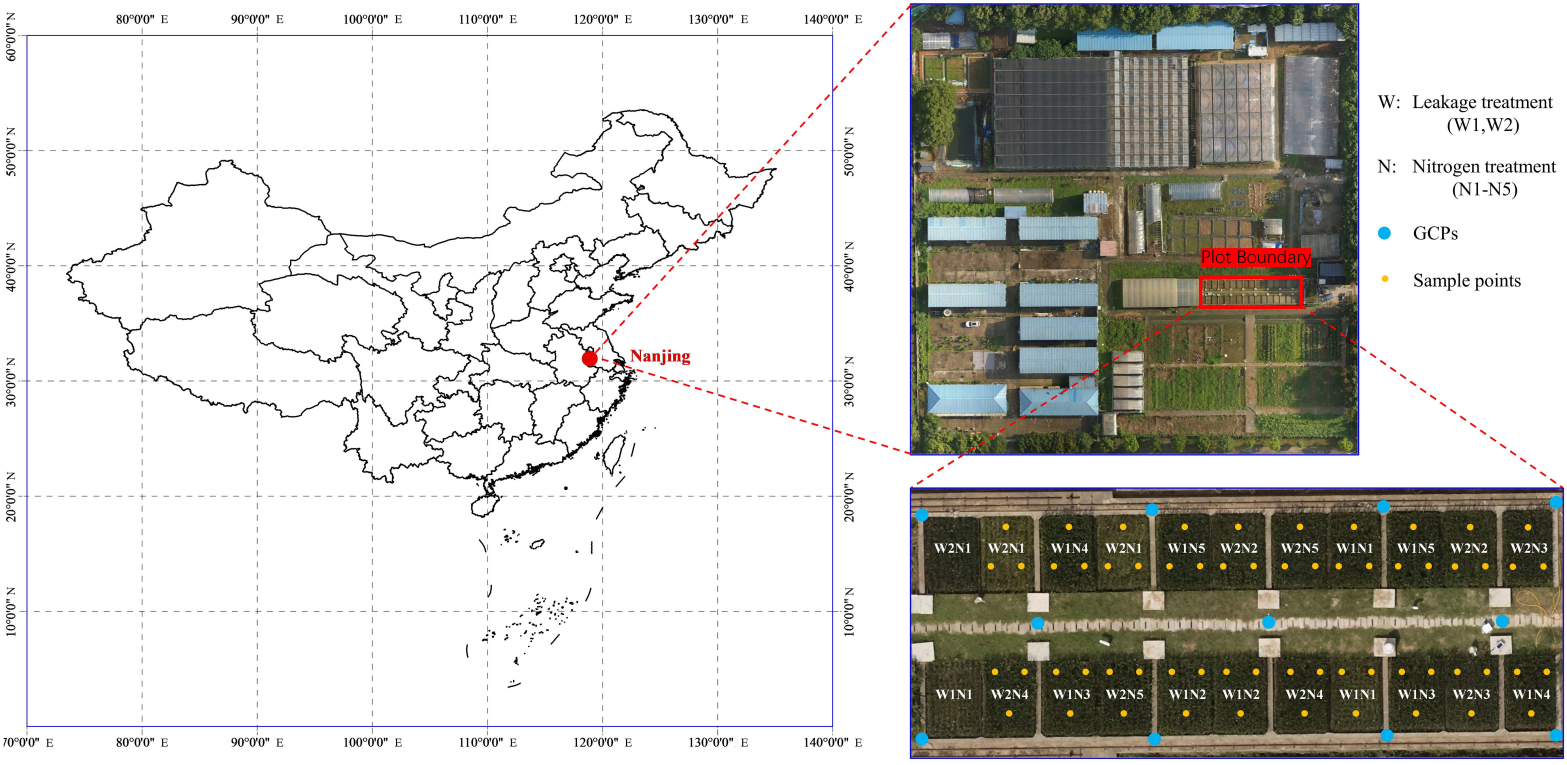
Study area and experiment treatments. W represents the leakage treatments (including W1: 3mm/day and W2: 5mm/day); N represents the nitrogen treatments (including N1-N5: 0, 150, 225, 300 and 375 kg/ha total pure nitrogen, respectively); GCP is abbreviation of gourd control points for geometric correction; Ground measurements in each sample point were averaged from 3 representative plants.

N represents the nitrogen treatments (including N1-N5: 0, 150, 225, 300 and 375 kg/ha total pure nitrogen, respectively)

In the published article, there was an error. The nitrogen application of N2–N5 fertilizer treatment levels was incorrectly written.

A correction has been made to **2 Materials and methods**, *2.1 Study area*, paragraph 1. This sentence previously stated:

“five nitrogen fertilizer levels (N1-N5: 0, 75, 150, 225, and 300 kg/ha total pure nitrogen)”

The corrected sentence appears below:

“five nitrogen fertilizer levels (N1–N5: 0, 150, 225, 300 and 375 kg/ha total pure nitrogen)”

The authors apologize for these errors and state that this does not change the scientific conclusions of the article in any way. The original article has been updated.

